# Static Magnetic Field Effects on Impaired Peripheral Vasomotion in Conscious Rats

**DOI:** 10.1155/2013/746968

**Published:** 2013-12-17

**Authors:** Shenzhi Xu, Hideyuki Okano, Masaaki Nakajima, Naoya Hatano, Naohide Tomita, Yoshito Ikada

**Affiliations:** ^1^Department of Mechanical Engineering and Science, Graduate School of Engineering, Kyoto University, Kyoto 606-8502, Japan; ^2^Research Section for Magnetics, Product Development Department, Development Division, PIP Company, Osaka 540-0011, Japan; ^3^Research Center for Frontier Medical Engineering, Chiba University, Chiba 263-8522, Japan; ^4^Department of Physical Therapy, School of Health Science and Social Welfare, Kibi International University, Okayama 716-8508, Japan; ^5^Department of Medical Engineering, Nara Medical University, Nara 634-8522, Japan

## Abstract

We investigated the SMF effects on hemodynamics in the caudal artery-ligated rat as an in vivo ischemia model using noninvasive near-infrared spectroscopy (NIRS) combined with power spectral analysis by fast Fourier transform. Male Wistar rats in the growth stage (10 weeks old) were randomly assigned into four groups: (i) intact and nonoperated cage control (*n* = 20); (ii) ligated alone (*n* = 20); (iii) ligated and implanted with a nonmagnetized rod (sham magnet; *n* = 22); and (vi) ligated and implanted with a magnetized rod (*n* = 22). After caudal artery ligation, a magnetized or unmagnetized rod (maximum magnetic flux density of 160 mT) was implanted transcortically into the middle diaphysis of the fifth caudal vertebra. During the experimental period of 7 weeks, NIRS measurements were performed in 3- , 5- , and 7-week sessions and the vasomotion amplitude and frequency were analyzed by fast Fourier transform. Exposure for 3–7 weeks to the SMF significantly contracted the increased vasomotion amplitude in the ischemic area. These results suggest that SMF may have a regulatory effect on rhythmic vasomotion in the ischemic area by smoothing the vasomotion amplitude in the early stage of the wound healing process.

## 1. Introduction

Increasing evidence of the importance of moderate-intensity magnetic field influence on in vivo physiological function has led to the consideration of medical applications of this knowledge [1–25]. For example, magnetic field therapy using moderate-intensity static magnetic fields (SMF; 1 mT–1 T) could be useful for circulatory diseases, including ischemic pain, inflammation, and hypertension, primarily due to the modulation of blood flow and/or blood pressure. In our previous studies, we have shown that subchronic exposure (3–12 weeks) to SMF (180 mT) accelerates bone recovery from operative invasion and ischemia induced by artery ligation [23, 24], or ovariectomy-induced osteoporosis [25], as indicated by the increased amount of bone mineral density (BMD). These studies suggested that SMF improved BMD probably due to recovery of microcirculation from bone ischemia. With regard to the effects of SMF on spectral properties of microcirculatory vasomotion, acute exposure (30–40 min) to SMF (30–223 mT) has been investigated for rats [14] and humans [20]. However, for prolonged exposure to SMF, there is no direct evidence. Focused on the subchronic exposure (3–7 weeks) to SMF (160 mT), the present study was undertaken to evaluate the effects of SMF on the vasomotion amplitude and frequency by fast Fourier transform.

## 2. Methods

### 2.1. Animals

Male Wistar rats of 10 weeks old and weighing 310–360 g were used for this study (Charles River Laboratories Japan Inc., Kyoto, Japan). All animal experiments were approved by the Kyoto University Animal Research Committee, and all the experimental procedures were conducted in accordance with the guiding principles of the *Guide for the Care and Use of Laboratory Animals* published by the National Institutes of Health (NIH Publication no. 85–23, revised in 1996).

### 2.2. Static Magnetic Fields

**Figure 1:**
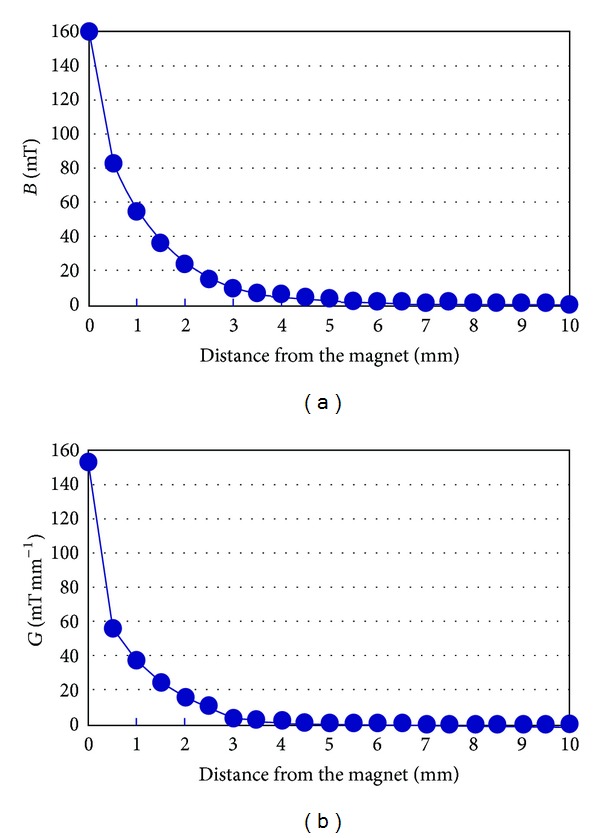
(a) Spatial distribution of the magnetic flux density values. (b) Spatial distribution of the magnetic gradient values.

A tapered rod was prepared from a samarium-cobalt alloy being designed as an elongated, narrow truncated cone (large diameter, 1.5 mm; small diameter, 1.1 mm; length, 6 mm; and weight, 61 mg), with or without magnetization of the rod. The entire surface was homogeneously coated with polyimide to prevent the release of metal ions from the alloys into the body. Every piece of magnet had a maximum magnetic flux density (*B*_max⁡_) of 160 mT and a maximum magnetic gradient (*G*_max⁡_) of 154 mT mm^−1^ (Figure 1). Both the *B*_max⁡_ and *G*_max⁡_ values were observed at the center of the large diameter surface (South pole) of the rod end of the magnet. The second highest values (*B*, 131 mT; *G*, 116 mT mm^−1^) were observed at the center of the small diameter surface (North pole), and the higher values were localized on both polar ends of the magnet perpendicular to the axis. The magnetic flux density was measured from the magnetic rods using a gaussmeter (Model 4048, Hall probe A-4048-002, Bell Technologies). All rods were sterilized by immersing in 0.5% dilute Hibitane solution for 30 min before implantation.

### 2.3. Experimental Procedure

After general intraperitoneal (i.p.) anesthesia with medetomidine (180 *μ*gkg^−1^) and midazolam (1.25 mgkg^−1^) in rats as described in our previous report [25], the dorsal caudal skin just over the fifth caudal vertebra was incised. The caudal artery was ligated with a suture and amputated at the fifth caudal vertebra. Caudal circulation just after the ligation was mainly supported by collateral vessels. The rod was vertically implanted into the fifth caudal vertebra in a similar manner as described in our previous reports [23, 24]. Briefly, for implantation, a 2 cm lateral skin incision was made, and, thereafter, the fifth caudal vertebra was exposed by blunt dissection of the caudal muscle. The periosteum was incised and pushed aside to drill a hole in the middle diaphysis of the fifth caudal vertebra from the lateral cortex to the medical cortex. The drill size was matched to that of the rod. A rod was implanted transcortically into the hole, applying a load of 500 g for 30 s using a digital force gauge. Thereafter, three layers of muscle, subcutaneous connective tissue, and skin were sutured. Spatial distribution of the magnetic flux density values in the caudal muscle (target tissue) was 160 mT or less (*B*_max_ = 160 mT).

**Figure 2:**
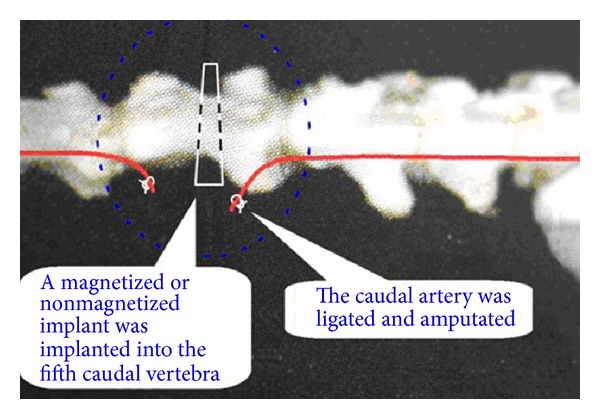
The caudal artery was ligated with a suture and amputated at the fifth caudal vertebra. Caudal circulation just after the ligation was mainly supported by collateral vessels. The rod was vertically implanted into the fifth caudal vertebra.

All rats were randomly assigned to one of four groups: (i) intact and nonoperated cage control (C); (ii) ligated alone (L); (iii) ligated and implanted with a nonmagnetized rod (sham magnet, L + S); and (vi) ligated and implanted with a magnetized rod (L + M). The sham magnet without magnetization was prepared for the rod used with the same size and materials as those of the magnet. The sham magnet was implanted in the same position of another rat to compare the effect with the magnet (Figure 2).

After the operation, two rats were housed together in one cage (LWH, 340 × 240 × 170 mm^3^). All animals had free access to water and standard pellet food and were kept under controlled lighting conditions (12 : 12 h light-dark cycle) at room temperature and relative humidity (25 ± 1°C and 55 ± 5%).

### 2.4. Measurements of Blood Circulation

After a 3-week healing period, peripheral blood circulation in the caudal muscle was estimated as tissue blood oxygen contents using noninvasive near-infrared spectroscopy (NIRS, BOM-L1TRW, Omega wave, Tokyo, Japan; during the healing period up to 3 weeks, the measurements were not carried out due to the suture-based intervention). The NIRS measurements of peripheral circulation in the ligated portion of caudal artery were performed in prone position under conscious condition (without anesthesia) in 3-, 5-, and 7-week sessions. The measurements in ligated alone group (L group) and control group (C group) were also conducted in the same manner. Thereafter, the vasomotion amplitude and frequency of the data were analyzed by Fourier transform. For the measurement, each rat was placed into an acrylic holder for a loose restraint. Prior to measurements, each rat was acclimated to the holder and tail cuff for at least 10 min without anesthesia. The measurement duration was 5 min.

NIRS is based on the weak absorption of near-infrared light by biological tissues and, in particular, in this measurement system, tissue levels of blood oxygen contents, that is, oxygenated hemoglobin (OxyHb), deoxygenated hemoglobin (DeoxyHb), and total hemoglobin (Total Hb), can be determined using three laser diodes (780, 810, and 830 nm) according to the Beer-Lambert law. The absorption coefficient of hemoglobin at each wavelength is based on the data reported by Matcher et al. [26]. In addition, the value of tissue blood oxygen saturation (StO_2_) can be calculated as:

(1) $$\begin{array}{c} \text{St}{\text{O}}_{2}=\frac{\text{OxyHb}}{\text{Total}\;\;\text{Hb}}\times 100\;\;\left(\%\right). \end{array}$$

It has been reported that NIRS parameters reflected the oxygenation consumption (VO_2_) in the local muscle by Kawaguchi et al. [27]. For taking the measurements, the laser transmitting probe and receiving detector were fixed on both lateral sides of the tail skin surface at the healed surgical incision site with a tail cuff without compression. Because the tail diameter was less than 10 mm, the fixed distance between the probe and detector was 10 mm, with a resulting penetration depth of 10 mm from the tail skin surface. The data were inputted into a personal computer at sampling frequency of 60 Hz. The analytical data were calculated one frame per one sec.

Statistical analysis was performed using one-way ANOVA followed by Bonferroni-Dunn post hoc test (StatView 5.0, SAS Institute Inc., USA) for each group of measurements (*P* < 0.05). All of the data were expressed as mean ± S.D.

## 3. Results

### 3.1. The Average Values of the Blood Circulation Parameters

In terms of the average values of the four blood circulation parameters (OxyHb, DeoxyHb, Total Hb, and StO_2_) in blood circulation in the caudal muscle, there was no significant difference between the groups in 3-, 5-, and 7-week sessions (data not shown).

### 3.2. The Average Values of the Vasomotion Amplitude and Frequency

**Figure 3:**
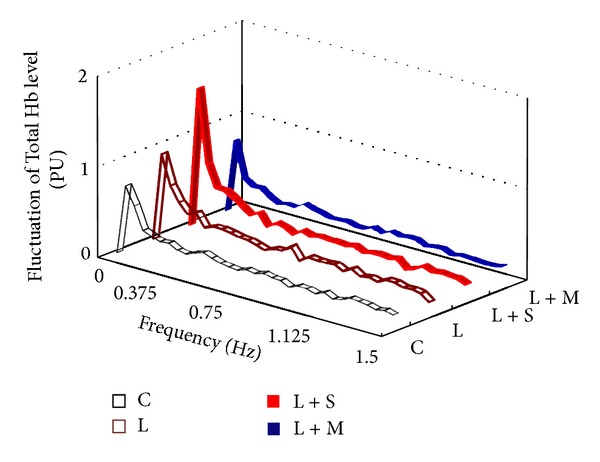
Relationship between vasomotion amplitude and frequency of the mean Total Hb level change in a 3-week exposure period. C, *n* = 20; L, *n* = 20; L + S, *n* = 22; L + M, *n* = 22. PU: perfusion unit.

**Figure 4:**
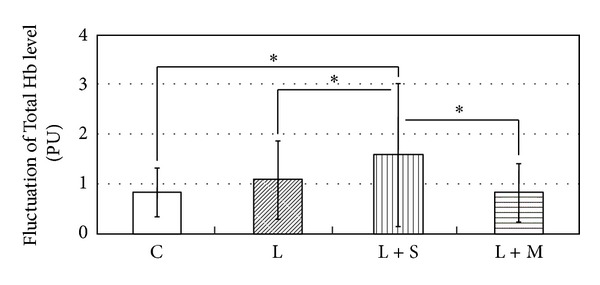
Fluctuation of Total Hb level at the frequency of 0.05 Hz 3 weeks after ligated and implanted operation. C, *n* = 20; L, *n* = 20; L + S, *n* = 22; L + M, *n* = 22. Mean ± S.D. PU: perfusion unit; *P < 0.05.

Fourier transform analysis indicated that the average values of vasomotion amplitude in four blood circulation parameters peaked at the frequency of 0.05 Hz, for example, the amplitude of Total Hb level change in a 3-week session (Figure 3). Therefore, at the frequency of 0.05 Hz, the average values of vasomotion amplitude were statistically compared between the groups. The average value of vasomotion amplitude for Total Hb in the L + S group was significantly larger than those for the C group, L group, and L + M group in a 3-week session (*P* < 0.05; Figure 4). Moreover, there was also no significant difference between the groups in 5- and 7-week sessions (data not shown). For other parameters, that is, DeoxyHb and StO_2_ values, there was no significant difference between the groups during the experimental period.

## 4. Discussion

Previous researches have shown that acute exposure (30–40 min) to SMF (30–223 mT) increased skin vasomotion amplitude [14, 20]. In resting skin blood flow in healthy young men, Yan et al. [20] found that SMF exposure at *B*_max⁡_ of 223 mT for 30 min induced a significant increase in vasomotion amplitude, mainly reflecting the intrinsic myogenic and endothelial-related metabolic activities, by placing a magnet to the center of the middle finger prominence and, after removal of the SMF, the vasomotion amplitude vanished gradually. In an animal (rat) model, Li et al. [14] reported significant enhancement of vasomotion amplitude, mainly reflecting the endothelial-related metabolic activity (0.01–0.05 Hz) in the skin stressed by pressure loading over the trochanter area upon exposure to an SMF at *B*_max⁡_ of 30 mT for 40 min. In their study, prolonged surface loading caused significant reduction of the endothelial-related metabolic activity and increased the myogenic activity, that is, induced a higher vascular tone in tissues that had been stressed as compared with the unstressed ones [14]. In contrast, SMF significantly increased the endothelial-dependent vasodilation and subsequently increased blood flow in the stressed skin [14]. The modulating effect of SMF on the vasomotion amplitude might be related to the vascular tone modified by prolonged compressive loading [14].

In contrast to the aforementioned previous studies [14, 20], we focused on examining the subchronic effects of moderate-intensity inhomogeneous SMF on peripheral hemodynamics. The present study indicated that SMF exposure for 3 weeks seems to have the tendency to modulate the vasomotion amplitude at 0.05 Hz in the range of endothelial-related metabolic activity for rats, and contract the increased vasomotion amplitude in the ischemic area, but did not induce significant change in any one of these parameters during the SMF exposure period of 3–7 weeks investigated. These results suggest that SMF may have a regulatory effect on rhythmic vasomotion in the ischemic area by smoothing the vasomotion amplitude, mainly reflecting the endothelial-related metabolic activity, in the early stage of the wound healing process. The physiological implication is that the smoothing or buffering of the vasomotion amplitude may play a key role on inherent hemodynamic control mechanisms for rhythmic vasomotion and endothelial-dependent vasodilation.

Other reports have shown that an SMF of strong intensity higher than a few Tesla has bioeffects [28–41]. For instance, an SMF of strong intensity with an extremely high magnetic gradient (*B*_max⁡_ of 8 T and *G*_max⁡_ of 400 T^2^/m) could induce some bioeffects on paramagnetic hemoglobin by magnetic attraction in a high gradient or diamagnetic hemoglobin by magnetic repulsion in a high gradient, retarding the mean blood velocity in peripheral circulation, partly due to the asymmetric distribution of red blood cells (RBC) with different magnetic susceptibilities and magnetically induced movement of diamagnetic water vapor at the skin surface, which may lead to a skin temperature decrease [36]. Moreover, it has been shown that RBC rotate and orient so that the concave surface is aligned parallel to strong uniform SMF due to magnetic torque [29]. Concerning the SMF effects on blood viscosity, different results were obtained, depending on the exposure conditions [34, 40, 41]. Haik et al. [34] reported an increase in viscosity of blood flowing parallel to inhomogeneous strong SMF at *B*_max⁡_ of 3, 5, and 10 T. Yamamoto et al. [40] also indicated an increased viscosity of both fully oxygenated and fully deoxygenated blood and greater increase in blood viscosity of deoxygenated blood relative to that of oxygenated blood in 1.5 T homogeneous SMF exposure for 36 min. In contrast, Tao and Huang [41] demonstrated that acute exposure to 1.33 T homogeneous SMF reduced blood viscosity when it was applied parallel to the flow direction (for an exposure duration of 1 min, short chains of RBC were formed; for an exposure duration of 12 min, long cluster chains of RBC were formed. This process could enable the RBC to pass through the blood vessels in a more streamlined fashion, thereby reducing the blood viscosity) [41]. These observations suggested that both inhomogeneous and homogeneous high intensity SMF (Tesla level) may modulate in vivo hemodynamics. However, the underlying mechanisms and physiological consequences have not yet been fully understood.

Because our applied SMF at *B*_max⁡_ of 160 mT was much lower in the magnetic force compared with the SMF of several Tesla, different plausible mechanisms might exist between them, such as through a Ca^2+^-calmodulin-regulated NO-cGMP signaling pathway. Takeshige and Sato [1] suggested a mechanism of SMF action for the promotion of hemodynamic responses in which an SMF at *B*_max⁡_ of 130 mT might inhibit acetylcholinesterase (AChE). Recovery of circulation is assumed to be partly due to the enhanced release of acetylcholine (ACh) by the SMF exposure, activating the cholinergic vasodilator nerve endings innervated to the muscle artery [1]. The inhibitory effect of SMF on AChE was also observed in the magnetic flux density of 0.8 mT or more [42]. In addition, it is also suggested that an SMF at *B*_max⁡_ of 5.5 mT should have a potential to counteract the action of a nitric oxide synthase (NOS) inhibitor L-NAME, presumably via increased endogenous ACh release [43, 44]. The increased (upregulated) effect of a 120 *μ*T SMF on endothelial nitric oxide synthase (eNOS) expression was also confirmed in human umbilical vein endothelial cells (HUVEC) [45].

In our study, SMF did not cause any significant change in DeoxyHb during the experimental period. In contrast, more recently, Muehsam et al. [44] found that exposure for 10–30 min to SMF of *B*_max⁡_ 186 mT resulted in more rapid Hb deoxygenation, using a reducing agent dithiothreitol in an in vitro cell-free preparation. Thus, in their study, SMF significantly increased the rate of Hb deoxygenation occurring several minutes to several hours after the end of SMF exposure [46]. The observation that SMF pretreatment of Hb alone or of the deoxygenation solution itself failed to yield a significant effect suggests that SMF exposure acted upon the interaction of Hb with the deoxygenation solution [46]. With regard to the mechanism, they speculated that these SMF modalities modified protein/solvation structure in a manner that altered the energy required for deoxygenation [46]. They further speculated that the mT-range SMF could induce the action of the Lorentz force on charges bound at the protein/water interface based on the Lorentz-Langevin model for weak magnetic field bioeffects [47, 48]. This model suggested that weak exogenous AC/DC magnetic fields can act on an ion/ligand bound in a molecular cleft based upon the assumption that the receptor molecule is able to detect the Larmor trajectory of an ion or ligand within the binding site. To date, however, there is insufficient direct experimental evidence pertaining to this model. Further studies are required to better understand the mechanisms of SMF bioeffects on hemodynamic function. Spectral analysis would be useful to examine these effects in more detail with a view of hemodynamics.

## 5. Conclusion

Exposure for 3–7 weeks to the SMF at *B*_max⁡_ of 160 mT and *G*_max⁡_ of 154 mT mm^−1^ significantly contracted the increased vasomotion amplitude in the ischemic area. It seems possible that SMF may have a regulatory effect on rhythmic vasomotion in the ischemic area by smoothing the vasomotion amplitude in the early stage of the wound healing process.
